# Metabolizable energy, nitrogen balance, and ileal digestibility of amino acids in quality protein maize for pigs

**DOI:** 10.1186/2049-1891-5-26

**Published:** 2014-05-07

**Authors:** Gerardo Mariscal-Landín, Tércia Cesária Reis de Souza, Ericka Ramírez Rodríguez

**Affiliations:** 1 CENID Fisiología, Instituto Nacional de Investigaciones Forestales Agrícolas y Pecuarias, km 1 Carretera a Ajuchitlán, 76280 Querétaro, México; 2 Facultad de Ciencias Naturales, Universidad Autónoma de Querétaro, México, Av. de las Ciencias s/n, 76230 Querétaro, México

**Keywords:** Amino acids, Energy balance, Maize, Nitrogen balance, Pigs

## Abstract

**Background:**

To compare the nutritional value and digestibility of five quality protein maize (QPM) hybrids to that of white and yellow maize, two experiments were carried out in growing pigs. In experiment 1, the energy metabolizability and the nitrogen balance of growing pigs fed one of five QPM hybrid diets were compared against those of pigs fed white or yellow maize. In experiment 2, the apparent and standardized ileal digestibility (AID and SID, respectively) of proteins and amino acids from the five QPM hybrids were compared against those obtained from pigs fed white and yellow maize. In both experiments, the comparisons were conducted using contrasts.

**Results:**

The dry matter and nitrogen intakes were higher in the pigs fed the QPM hybrids (*P* < 0.05) than in the pigs fed white or yellow maize. Energy digestibility (*P* < 0.001) and metabolizability (*P* < 0.01) were higher in the pigs fed the white and yellow maize diets than in those fed the QPM diets. The AID of lysine was higher (*P* < 0.01) in the QPM diets than in the white and yellow maize. The AIDs of leucine, isoleucine, valine, phenylalanine, and methionine were lower in the QPM diets than those of maize (white and yellow) (all *P* < 0.05). Maize (white and yellow) had greater SIDs of leucine, isoleucine, valine, phenylalanine, glutamic acid, serine, alanine, tyrosine, and proline (*P* < 0.05).

**Conclusions:**

Based on these results, it was concluded that QPM had a lower metabolizable energy content and a higher amount of digestible lysine than normal maize.

## Background

Maize (*Zea mays*) is the most widely harvested cultivar in the world, and it is often used as the principal source of energy for pigs. It is also an important source of protein and amino acids in finishing pigs [1]. There are varieties of maize, other than yellow maize, that contain different nutrient concentrations [2]. Owing to these differences in nutrient density and/or composition, new types of maize such as quality protein maize (**QPM**) may offer nutritional advantages over the conventional yellow maize varieties.

Maize protein is deficient in the amino acids, lysine and tryptophan, which limits its value for monogastric animals [3]. Mertz et al. [4] first reported the mutant maize called Opaque 2 in 1963, which has a higher content of these amino acids. However, maize Opaque 2 had a soft endosperm that made it susceptible to pests and crop storage problems, for which its production was ceased. Subsequent conventional breeding efforts by the International Maize and Wheat Improvement Centre (CIMMYT) generated numerous cultivars with improved agronomic characteristics, collectively referred to as QPM [5], a type of maize with a hard endosperm rich in lysine and tryptophan due to a change in the ratio of 19- and 22-kD α zeins, an increment of 27 kD γ zein [6], and a non zein protein called elongation factor 1α (**EF-1 α**) [7]. The substantial reduction in synthesis of α-zeins results in smaller, less numerous protein bodies and a concomitant increase in non-zein endosperm proteins [3].

The metabolizable energy (**ME**) and amino acid (**AA**) digestibility of yellow maize have been extensively investigated and summarized in previous publications [8,9]. This allows for the accurate formulation of yellow maize-based diets for pigs to meet ME and digestible AA requirements. However, there is currently limited information about the nutritive value of QPM.

Therefore, the first aim of the current study was to compare the energy metabolizability and nitrogen balance of QPM to those of yellow and white maize, and the second aim was to compare the apparent and standardized ileal digestibility (**AID** and **SID**, respectively) of protein and AAs in QPM hybrids to those of yellow and white maize.

## Material and methods

This study was approved by the Scientific Associate Technical Group Committee of CENID Physiology. Two experiments were conducted at the experimental farm of CENID-Physiology (INIFAP, México). The experimental animals were treated according to the guidelines of the International Guiding Principles for Biomedical Research Involving Animals [10] and the Official Mexican Standard for production, care and use of laboratory animals [11].

### Raw materials

The following QPM hybrids were evaluated: 538Ta, 537Ta, 537Ig, 334Ce, and QPM1. Evaluation was also conducted on one white and one yellow maize obtained from a commercial supplier in the state of Guanajuato, Mexico. The chemical composition of these materials is shown in Table 1.

**Table 1:** **Chemical composition of maize (g/kg)**^**1**^

	**QPM**^**2**^					**Maize**	
**Nutrient, g /kg**	**QPM1**	**334Ce**	**537Ta**	**537Ig**	**538Ta**	**White**	**Yellow**
Dry Matter	910.7	911.4	893.0	909.0	911.2	911.0	910.8
Protein	85.0	84.0	86.0	85.0	87.0	80.0	75.0
NDF	114.0	91.0	102.0	84.0	89.0	104.0	94.0
ADF	48.0	39.0	40.0	38.0	35.0	34.0	27.0
Ether Extract	34.0	34.0	47.0	39.0	52.0	39.0	37.0
Ash	17.0	14.0	16.0	17.0	17.0	11.0	12.0
Gross Energy, MJ/kg	16.7	16.6	16.4	16.0	16.6	16.5	16.5
Amino acids							
Alanine	5.1	4.9	4.9	5.0	4.2	5.9	5.6
Arginine	5.9	3.5	5.3	5.3	4.9	3.8	3.6
Aspartic acid	6.7	4.6	6.5	6.4	5.4	5.4	5.3
Cystine	3.5	3.5	2.4	3.5	3.4	3.0	3.3
Glutamic acid	14.1	12.6	13.6	14.4	11.4	15.3	14.2
Glycine	4.1	2.9	3.9	4.0	3.5	3.1	3.0
Histidine	3.5	2.7	3.4	2.5	2.6	2.5	2.1
Isoleucine	2.6	2.0	2.5	2.4	2.1	2.6	2.5
Leucine	7.3	7.7	7.1	7.3	5.8	9.6	9.1
Lysine	3.1	2.7	2.9	2.7	2.7	2.0	2.0
Methionine	1.8	1.8	2.2	2.3	2.3	1.5	2.1
Phenylalanine	3.9	3.1	3.4	3.9	2.9	4.2	3.9
Proline	8.4	7.4	9.4	8.0	7.9	9.2	8.9
Serine	3.7	3.1	3.6	3.9	3.1	3.7	3.5
Threonine	3.3	2.4	3.0	3.5	2.7	2.8	2.6
Tyrosine	2.6	2.2	2.4	2.2	2.1	2.6	2.5
Valine	4.7	3.1	5.4	4.4	4.6	3.9	4.3

^1^As fed basis.

^2^Quality Protein Maize.

### Energy metabolizability and nitrogen balance (Experiment 1)

Four consecutive groups of seven Landrace x Duroc pigs with a mean weight of 61.2 ± 2.6 kg were used (28 pigs in total, four replicates by treatment). The pigs were placed in individual metabolic cages equipped with a self-feeder and a low-pressure drinking nipple connected to a watering system that controlled the water supply. Screens were placed under the floors, which allowed for total collection of feces and urine. The room temperature ranged from 19–22°C.

The pigs were fed twice daily at 0800 h and 1800 h. The experimental diets (Table 2) were prepared with one QPM or one maize (white or yellow) as the sole protein and energy source. The diets contained calcium carbonate, dicalcium phosphate, salt, and premixed vitamins and minerals. Chromic oxide (3 g/kg of feed) was included as an indigestible marker. The feed intake of the pigs was 2.5 times their digestible energy (**DE**) requirement for maintenance (460 kJ/kg BW^0.75^[12]) as recommended by Adeola [13] for pigs weighing >50 kg. The water container in each metabolic cage was filled just before each meal to restrict water intake to 2.5 L/kg of dry matter (**DM**) intake [13].

**Table 2:** **Composition of the experimental diets (g/kg): experiment 1**^**1**^

**Ingredient, g/kg**	**QPM**^**2**^					**Maize**	
	**QPM1**	**334Ce**	**537Ta**	**537Ig**	**538Ta**	**White**	**Yellow**
QPM1	967.5						
334Ce		967.5					
537Ta			967.5				
537Ig				967.5			
538Ta					967.5		
White						967.5	
Yellow							967.5
Calcium Phosphate	18.5	18.5	18.5	18.5	18.5	18.5	18.5
Calcium Carbonate	5.5	5.5	5.5	5.5	5.5	5.5	5.5
Salt	3.5	3.5	3.5	3.5	3.5	3.5	3.5
Minerals^3^	1.0	1.0	1.0	1.0	1.0	1.0	1.0
Vitamins^4^	1.0	1.0	1.0	1.0	1.0	1.0	1.0
Chromic oxide	3.0	3.0	3.0	3.0	3.0	3.0	3.0
Chemical composition^5^							
Dry Matter	906.6	903.3	891.1	904.6	901.4	905.6	904.8
Protein	82.2	81.3	83.2	82.2	84.2	77.4	72.6
Gross Energy, MJ/kg	16.5	16.2	16.1	15.9	16.1	16.0	16.0

^1^As fed basis.

^2^Quality Protein Maize.

^3^Furnished by kg of feed: Cl 1.65 g, Na 0.87 g, Cu 7.7 mg, Fe 89.25 mg, Mn 19.98 mg, Se 0.087 mg, I 0.053 mg.

^4^Furnished by kg of feed: Vitamin A 6,600 IU, D 660 IU, E 100 IU, Choline 350 mg, Niacin 54 mg, Pantothenic acid 13.15 mg, Riboflavin 2.2 mg, B_12_ 36 μg.

^5^Analyzed values, on an as fed basis.

The experimental period lasted for 10 d for each consecutive group: 5 d for adaptation and 5 d for the total collection of feces and urine. The feces were frozen and kept at -20°C. At the end of the experimental period, the feces were defrosted and homogenized to obtain 10% of the weight as a final sample for lyophilizing. Urine was collected via funnels underneath the urine collection tray. This collection system included a glass wool mat to avoid contamination with feed or feces. To reduce urine pH and avoid NH_3_ volatilization, 40 mL of HCl 6 mol/L were added to each urine container twice a day. The urine was removed twice a day, weighed, and filtered again through four layers of cheesecloth into a clean container. Then a 5% aliquot was taken and kept at -20°C until analysis. Three urine subsamples of 20 mL per pig were lyophilized to measure energy [14].

### Ileal digestibility (Experiment 2)

Seven Landrace x Duroc pigs with a mean weight of 62.3 ± 4.9 kg at the time of data collection were used. When the pigs weighed 45 kg, a T-cannula was fitted at the terminal ileum [15]. After surgery, the pigs were placed in individual metabolism cages that included a self-feeder and a low-pressure drinking nipple. The room temperature ranged from 19–22°C.

The post-operative period lasted for 21 d. During this period, the pigs were fed a grower diet (160 g of CP/kg) twice daily at 0800 h and 1800 h. The amount fed was increased 100 g/d until the level of intake was the same as that prior to surgery.

During the experimental period, the pigs received one of the experimental diets (Table 3). The diets were formulated using maize as the sole source of dietary protein. To avoid the effect of the level of the dietary protein on protein apparent digestibility [16], maize starch was used to ensure that the experimental diets consisted of the same protein level despite the different protein levels of the maize. All experimental diets contained calcium carbonate, dicalcium phosphate, and salt. To reduce dust, 20 g/kg of maize oil was included. Vitamins and minerals exceeded the NRC [8] requirements. Chromic oxide (3 g/kg of feed) was included as an indigestible marker. The feed intake of the pigs was 2.5 times their digestible energy (DE) requirement for maintenance, 460 kJ/kg BW^0.75^[12]. The animals had free access to water.

**Table 3:** **Composition of the experimental diets (g/kg): experiment 2**^**1**^

**Ingredient, g/kg**	**QPM**^**2**^					**Maize**	
	**QPM1**	**334Ce**	**537Ta**	**537Ig**	**538Ta**	**White**	**Yellow**
QPM1	845.5						
334Ce		845.5					
537Ta			825.5				
537Ig				835.5			
538Ta					816.5		
White						887.5	
Yellow							947.5
Maize starch	102.0	102.0	122.0	112.0	131.5	60.0	
Calcium Phosphate	18.5	18.5	18.5	18.5	18.5	18.5	18.5
Calcium Carbonate	5.5	5.5	5.5	5.5	5.5	5.5	5.5
Maize oil	20.0	20.0	20.0	20.0	20.0	20.0	20.0
Salt	3.5	3.5	3.5	3.5	3.5	3.5	3.5
Minerals^3^	1.0	1.0	1.0	1.0	1.0	1.0	1.0
Vitamins^4^	1.0	1.0	1.0	1.0	1.0	1.0	1.0
Chromic oxide	3.0	3.0	3.0	3.0	3.0	3.0	3.0
Chemical composition^5^							
Dry matter	905.6	901.3	894.1	864.2	899.2	906.5	902.8
Protein	71.1	70.8	70.6	71.0	71.3	70.5	71.6
Gross Energy, MJ/kg	15.8	15.7	15.6	15.2	15.7	15.7	15.6
Alanine	4.70	4.81	4.45	4.32	4.01	5.55	6.08
Arginine	5.36	3.47	4.86	4.57	4.70	3.59	3.93
Aspartic acid	6.10	4.54	5.83	5.59	5.04	5.04	5.69
Cystine	3.22	3.47	2.19	3.13	3.25	2.82	3.53
Glutamic acid	12.87	12.47	12.38	12.62	10.85	14.43	15.41
Glycine	3.71	2.85	3.56	3.56	3.33	2.90	3.24
Histidine	3.22	2.67	3.08	2.54	2.48	2.39	2.36
Isoleucine	2.39	1.96	2.19	1.61	1.96	2.39	2.65
Leucine	6.60	7.57	6.39	6.10	5.55	9.05	9.81
Lysine	2.80	2.67	2.59	2.54	2.48	1.88	2.16
Methionine	1.65	1.16	2.02	1.10	1.45	1.45	2.26
Phenylalanine	3.55	3.12	3.08	2.80	2.82	3.93	4.22
Proline	6.35	6.14	8.50	4.91	7.52	8.71	9.62
Serine	3.38	3.03	3.24	3.47	2.90	3.50	3.73
Threonine	2.97	2.40	2.75	3.39	2.56	2.65	2.85
Tyrosine	2.39	2.23	2.19	2.12	1.96	2.48	2.65
Valine	4.29	3.03	4.86	2.71	4.36	3.59	4.71

^1^As fed basis.

^2^Quality Protein Maize.

^3^Furnished by kg of feed: Cl 1.65 g, Na 0.87 g, Cu 7.7 mg, Fe 89.25 mg, Mn 19.98 mg, Se 0.087 mg, I 0.053 mg.

^4^Furnished by kg of feed: Vitamin A 6,600 IU, D 660 IU, E 100 IU, Choline 350 mg, Niacin 54 mg, Pantothenic acid 13.15 mg, Riboflavin 2.2 mg, B_12_ 36 μg.

^5^Analyzed values, on an as fed basis.

The four experimental periods lasted seven d each: 5 d for adaptation and 2 d for digesta collection. Ileal digesta were collected continuously over a period of 10 h (0800 h–1800 h) using plastic bags (11 cm × 5 cm) that contained 10 mL of a 0.2 mol/L HCl solution to inhibit bacterial activity and were attached to the cannula using a rubber band. When the collecting bags were full, the ileal digesta was transferred to a container and frozen at -20°C until lyophilisation.

At the end of the experiment, narcosis was induced by CO_2_ inhalation, followed by euthanasia by exsanguination. A post-mortem examination for fistula along the length of the small intestine was performed to verify its integrity.

### Chemical analysis

The raw materials, diets, digesta, and feces were ground using a laboratory mill (Arthur H. Thomas Co., Philadelphia, PA) to pass through a 0.5 mm mesh sieve. DM and nitrogen analysis methods, 934.01 and 976.05 [17], respectively, were performed. Chromium analysis was performed as described by Fenton and Fenton [18]. Gross energy was estimated using an adiabatic bomb calorimeter (model 1281, Parr, Moline, IL). Samples from the raw materials, diets, and digesta were hydrolyzed at 110°C for 24 h in 6 mol/L HCl to use in AA analysis method 994.12 [17]. For methionine and cystine analyses, oxidation with performic acid was carried out before acid hydrolysis [17]. Tryptophan was not estimated. AA analysis was performed using reverse phase HPLC (1100 HPLC Hewlett Packard), according to Henderson *et al.*[19]. Nitrogen in the liquid urine was estimated according to AOAC [17] method 976.05. Energy in the lyophilized urine was estimated according to Le Bellego [14].

### Calculations

The AID or apparent total tract digestibility (**ATTD**) was estimated using the equation proposed by Fan and Sauer [20]:

$$\mathrm{AID}=\left[1-\left(\left(\mathrm{ID}\times \mathrm{AF}\right)/\left(\mathrm{AD}\times \mathrm{IF}\right)\right)\right]\times 100$$

where AID = apparent digestibility (ileal or total tract) of a nutrient in the diet, **ID** = concentration of the marker in the diet (mg/kg DM), **AF** = concentration of the nutrient in ileal digesta or feces (mg/kg DM), **AD** = concentration of the nutrient in the diet (mg/kg DM), and **IF** = concentration of the marker in the ileal digesta or feces (mg/kg DM).

The SID was estimated using the formula proposed by Furuya and Kaji [21]:

$$\mathrm{SID}=\left[\mathrm{AID}+\left(\mathrm{Endogenous}/\mathrm{Dietary}\;\mathrm{Content}\right)\right]\times 100$$

where Endogenous = endogenous losses of a nutrient in mg/kg DM intake, and Dietary content = amount of nutrient consumed in mg/kg DM intake. The calculations were performed using endogenous values reported by Mariscal-Landin and Reis de Souza [22].

The ME was obtained using the formula proposed by Adeola [13]:

$$\mathrm{ME}=\left[\left(\mathrm{GE}-\mathrm{FE}-\mathrm{UE}\right)/\mathrm{GE}\right]\times 100$$

where ME = metabolizable energy in MJ/d, **GE** = gross energy intake in MJ/d, **FE** = fecal energy output in MJ/d, and **UE** = urine energy output in MJ/d.

### Statistical analyses

#### *Experiment 1*

Data were analyzed as a randomized complete block design [23] with four blocks of seven animals each and using the GLM procedure in SAS v9.2 [24]. The variables were the DM amount, nitrogen intake, energy intake, the amount of nitrogen and energy excreted in feces and urine, the apparent total tract digestibility of DM, nitrogen, and energy, nitrogen balance, and energy metabolizability. An alpha value of 0.05 was used to assess significance.

#### *Experiment 2*

Data were analyzed using a Latin square with additional columns, or a 4 × 7 “Youden square” [23], which included seven animals, seven treatments, and four experimental periods. The experimental variables were the protein and amino acids AID and SID of the maize. Data were analyzed using the GLM procedure in SAS v9.2 [24]. An alpha value of 0.05 was used to assess significance.

In both experiments, the means were compared using Duncan’s method and the QPM (QPM1, 334Ce, 537Ta, 537Ig, or 538Ta) and normal maize (white or yellow) were compared using contrasts [23].

## Results

### Energy metabolizability and nitrogen balance

#### *Energy metabolizability, QPM vs normal maize*

Energy intake was similar (*P >* 0.05) between the treatments: 29.5 MJ/d of QPM or normal maize. Apparent total tract digestibility was lower in the pigs fed QPM (89.8) than in those fed normal maize (91.0, *P <* 0.001). While UE was similar (0.58 MJ/d, *P >* 0.05) between all the treatments, the metabolizability of energy was lower in the pigs fed QPM (87.9) than in those fed normal maize (88.9) (Table 4).

**Table 4:** **Energy metabolizability and nitrogen balance: experiment 1**^**1**^

**Traits**	**QPM**^**2**^					**Maize**			**Contrast**		
	**QPM1**	**334Ce**	**537Ta**	**537Ig**	**538Ta**	**White**	**Yellow**	**SEM**^**3**^	**QPM**	**Normal**	**Prob**^**4**^
Dry Matter, kg/d											
Intake	1.67^a^	1.66^a^	1.66^a^	1.68^a^	1.64^a^	1.66^a^	1.55^b^	0.010	1.66	1.60	0.05
Energy, MJ/d											
Intake	29,9^a^	29.7^a^	29.7^a^	30.2^a^	29.5^a^	29.7^a^	27.7^b^	0.17	29.8	28.7	0.63
In feces	3.1^a^	3.2^a^	3.0^a^	2.9^ab^	2.9^ab^	2.6^b^	2.6^b^	0.04	3.0	2.6	0.001
Digestible	26.8^a^	26.5^a^	26.7^a^	27.3^a^	26.5^a^	27.1^a^	25.1^b^	0.16	26.7	26.1	0.01
In Urine	0.50	0.58	0.53	0.60	0.62	0.60	0.61	0.013	0.57	0.60	0.21
Metabolizable	26.3^a^	25.9^a^	26.2^a^	26.7^a^	25.9^a^	26.5^a^	24.5^b^	0.16	26.2	25.5	0.01
Nitrogen, g/d											
Intake	22.4^b^	24.6^a^	24.5^a^	24.6^a^	24.4^a^	22.0^b^	22.1^b^	0.13	24.0	22.0	0.001
In feces	3.6	4.1	3.8	3.7	3.8	3.4	3.5	0.08	3.82	3.46	0.32
Digestible	18.7^b^	20.5^a^	20.6^a^	20.4^a^	20.5^a^	18.5^b^	18.6^b^	0.35	20.1	18.6	0.001
In Urine	10.2^a^	12.6^b^	13.2^b^	11.9^b^	12.0^b^	12.1^b^	12.2^b^	0.21	11.97	12.2	0.74
Retained	8.5^a^	7.9^ab^	7.5^ab^	8.5^a^	8.5^a^	6.5^ab^	6.4^b^	0.24	8.18	6.44	0.19
Dry matter, MJ/kg											
Digestible energy	16.11^cd^	15.96^d^	16.12^bcd^	16.29^ab^	16.18^abc^	16.34^a^	16.22^abc^	0.007	16.13	16.28	0.01
Metabolizable energy	15.59^ab^	15.38^b^	15.59^ab^	15.70^a^	15.55^ab^	15.74^a^	15.61^ab^	0.009	15.56	15.68	0.09
Coefficient											
DM Digestibility	88.7^c^	88.2^c^	88.9^c^	90.1^ab^	89.2^bc^	90.8^a^	90.2^a^	0.12	89.0	90.5	0.001
E Digestibility	89.6^bc^	89.1^c^	89.8^bc^	90.4^ab^	90.1^bc^	91.3^a^	90.6^ab^	0.14	89.8	91.0	0.001
Metabolizability	87.9^b^	87.2^b^	88.0^b^	88.4^ab^	88.0^b^	89.3^a^	88.4^ab^	0.15	87.9	88.9	0.01
N Digestibility	83.7	83.3	84.3	84.7	84.3	84.3	84.2	0.30	84.1	84.3	0.72
N retention as percentage of											
Intake^5^	38.2^a^	32.1^ab^	30.5^ab^	35.2^ab^	35.0^ab^	29.6^ab^	28.8^b^	1.01	34.2	29.2	0.28
Absorbed^6^	45.7^a^	38.5^ab^	36.1^ab^	41.6^ab^	41.3^ab^	35.0^b^	34.2^b^	1.14	40.3	34.6	0.20

^1^All data are reported on a DM basis.

^2^Quality Protein Maize.

^3^Standard error of the mean.

^4^Contrast Probability.

^5^(N retained/N intake)*100.

^6^(N retained/N absorbed)*100.

^abc^different letters in the same line differ (*P <* 0.05).

#### *Energy metabolizability, means comparison*

Energy intake was lower in the pigs fed yellow maize (27.7 MJ/d) than in the pigs fed white maize (29.8 MJ/d, *P <* 0.05). Apparent total tract digestibility was higher in the pigs fed white maize (91.3) and lower in the pigs fed 334Ce QPM (89.1, *P <* 0.05). The metabolizability of energy was higher in the pigs fed white maize (89.3) than in the pigs fed QPM1 (87.9), except for those fed QPM 537Ig (88.4; Table 4).

#### *Nitrogen balance, QPM vs normal maize*

The DM intake was higher in the pigs fed QPM, compared to those fed normal maize (1.66 vs 1.60 kg/d, *P* < 0.05), and the DM total tract digestibility was lower in the QPM pigs, compared to those fed normal maize (89.0 vs 90.5, *P* < 0.001). The daily nitrogen intake was higher in the pigs fed QPM (24.0 g/d) than in the pigs fed normal maize (22.0 g/d, *P* < 0.001); however, nitrogen digestibility was similar between the treatments (84.1, *P* > 0.05). Consequently, the digestible nitrogen intake was higher in the pigs fed QPM. Urinary nitrogen excretion was similar between treatments (12.0 g/d, *P* > 0.05). Nitrogen retention as a proportion of nitrogen intake or nitrogen absorption was also similar (*P* > 0.05) between the treatments (Table 4).

#### *Nitrogen balance, means comparison*

Although the nitrogen intake in the pigs fed QPM1 was lower (22.4 g/d) than in the pigs fed the other hybrid QPMs (24.5 g/d, *P* < 0.001) and similar to the pigs fed normal maize (22.0 g/d), the pigs fed QPM1 retained more nitrogen as a percentage of nitrogen intake (38.2%) or nitrogen absorption (45.7%) than the pigs fed yellow maize (28.8% and 34.2%, respectively, *P* < 0.05; Table 4).

### Ileal digestibility

#### *Apparent ileal digestibility, QPM vs normal maize*

The DM digestibility was lower in the pigs fed QPM (78.7) than in those fed normal maize (80.0, *P* < 0.05). The CP digestibility was similar in all of the diets (mean 73.0, *P* > 0.05).

Lysine digestibility was higher in the pigs fed QPM than in the pigs fed normal maize (*P* < 0.05; Table 5). The digestibility of leucine, isoleucine, phenylalanine, glutamic acid, alanine, tyrosine, proline, valine, serine, and methionine was lower in the QPM pigs than in those fed normal maize (all *P* < 0.05; Table 5).

**Table 5:** Apparent ileal digestibility of the maize: experiment 2

**Traits**	**QPM**^**1**^					**Maize**			**Contrast**		
	**QPM1**	**334Ce**	**537Ta**	**537Ig**	**538Ta**	**White**	**Yellow**	**SEM**^**2**^	**QPM**	**Normal**	**Prob**^**3**^
Dry Matter	77.5^c^	77.6^c^	81.9^a^	77.7^c^	78.6^bc^	80.9^ab^	79.1^abc^	0.33	78.7	80.0	0.05
Protein	72.0	74.3	73.4	71.4	74.3	71.8	74.1	0.32	73.1	72.9	0.77
Amino acids											
Alanine	73.2^b^	72.7^b^	71.6^b^	69.0^b^	70.6^b^	79.8^a^	80.1^a^	0.67	71.4	79.9	0.001
Arginine	82.1^a^	73.8^b^	82.3^a^	81.3^a^	85.5^a^	80.0^a^	82.7^a^	0.72	81.0	81.4	0.79
Aspartic acid	75.2	68.4	75.9	71.5	71.9	75.4	75.6	0.61	72.6	75.5	0.06
Cystine	72.9^c^	78.9^ab^	66.9^d^	74.9^bc^	78.0^abc^	75.1^bc^	80.9^a^	0.56	74.3	78.0	0.07
Glutamic acid	83.3^b^	83.1^b^	83.1^b^	81.5^b^	82.1^b^	86.9^a^	86.9^a^	0.29	82.6	86.9	0.001
Glycine	55.1^ab^	49.8^b^	58.9^a^	54.6^ab^	51.9^ab^	55.8^ab^	57.0^ab^	0.91	54.0	56.4	0.83
Histidine	87.2^ab^	83.8^bc^	88.3^a^	82.6^c^	85.4^abc^	86.1^abc^	84.8^abc^	0.45	85.4	85.4	0.93
Isoleucine	75.4^ab^	69.9^b^	74.3^ab^	60.2^c^	71.8^b^	79.4^a^	79.2^a^	0.66	70.3	79.3	0.001
Leucine	82.4^b^	83.2^b^	83.1^b^	80.5^b^	82.1^b^	89.0^a^	89.4^a^	0.43	82.3	89.2	0.001
Lysine	77.2^a^	76.7^a^	77.1^a^	73.8^ab^	75.1^ab^	69.0^b^	72.4^ab^	0.72	76.0	70.7	0.01
Methionine	80.5^b^	74.2^c^	85.5^a^	70.7^d^	75.5^c^	76.8^c^	84.3^a^	0.36	77.3	80.5	0.05
Phenylalanine	82.7^b^	80.6^bc^	81.9^b^	77.3^c^	82.6^b^	88.0^a^	87.6^a^	0.46	81.0	87.7	0.001
Proline	51.2^cd^	61.6^bc^	72.5^ab^	45.8^d^	61.5^bc^	71.6^ab^	80.3^a^	1.63	58.5	75.9	0.001
Serine	71.0^ab^	67.8^b^	72.0^ab^	70.4^ab^	69.4^b^	76.8^a^	76.6^a^	0.70	70.1	76.6	0.01
Threonine	60.4^ab^	54.6^b^	61.6^ab^	64.1^a^	60.1^b^	64.8^a^	62.4^ab^	0.96	60.1	63.6	0.39
Tyrosine	77.9^b^	76.8^b^	77.7^b^	73.8^b^	74.7^b^	83.7^a^	83.9^a^	0.57	76.2	83.8	0.001
Valine	77.6^a^	70.4^b^	81.7^a^	63.3^c^	80.9^a^	78.5^a^	82.8^a^	0.18	74.8	80.6	0.01

^1^Quality Protein Maize.

^2^Standard error of the mean.

^3^Probability of contrast.

^abcd^different letters in the same line differ (*P <* 0.05).

#### *Apparent ileal digestibility, means comparison*

In general, QPM 537Ig had the lowest digestibility, except for that for arginine, threonine, serine, and glycine; for these AAs, QPM 334Ce had the lowest digestibility (Table 5). The white maize diet demonstrated lower AID of the sulphur AAs than the yellow maize diet. The digestibility of leucine, alanine, and tyrosine was lower in all of the QPM diets than in the normal maize diets (Table 5).

#### *Standardized ileal digestibility, QPM vs normal maize*

The digestibility of CP, lysine, arginine, histidine, methionine, threonine, aspartic acid, glycine, and cystine was similar between QPM and normal maize (all *P* > 0.05; Table 6). The digestibility of glutamic acid, tyrosine, leucine, isoleucine, phenylalanine, alanine, valine, serine, and proline was lower in the QPM diets than in the normal maize diets (all *P* < 0.05; Table 6).

**Table 6:** Standardized ileal digestibility of the maize: experiment 2

**Traits**	**QPM**^**1**^					**Maize**			**Contrast**		
	**QPM1**	**334Ce**	**537Ta**	**537Ig**	**538Ta**	**White**	**Yellow**	**SEM**^**2**^	**QPM**	**Normal**	**Prob**^**3**^
Protein	86.7	88.0	88.2	85.7	88.2	86.9	88.1	0.32	87.4	87.5	0.98
Amino acids											
Alanine	80.7^ab^	80.0^bc^	79.4^c^	77.1^c^	79.4^c^	86.1^a^	85.8^ab^	0.66	79.3	86.0	0.01
Arginine	87.9^ab^	82.8^b^	88.8^ab^	88.2^ab^	92.2^a^	88.8^ab^	90.8^a^	0.72	88.0	89.8	0.65
Aspartic acid	85.2	81.9	86.4	82.4	84.0	87.5	86.4	0.61	84.0	86.9	0.06
Cystine	77.1^cd^	82.8^ab^	73.1^c^	79.2^bc^	82.1^abc^	79.9^abc^	88.7^a^	0.56	78.9	82.3	0.10
Glutamic acid	88.7^b^	88.7^b^	88.7^b^	87.1^b^	88.6^b^	91.7^a^	91.4a	0.28	88.3	91.6	0.001
Glycine	73.8	74.2	78.3	74.2	72.7	79.7	78.4	0.91	74.6	79.0	0.26
Histidine	92.0^ab^	89.5^ab^	93.3^a^	88.6^b^	91.5^ab^	92.5^ab^	91.3^ab^	0.46	91.0	91.9	0.39
Isoleucine	86.3^ab^	83.3^b^	86.3^ab^	76.4^c^	85.0^ab^	90.3^a^	89.0^ab^	0.66	83.4	89.7	0.01
Leucine	89.2^bc^	89.1^bc^	90.2^abc^	87.9^c^	90.2^abc^	94.0^a^	94.0^a^	0.44	89.3	94.0	0.01
Lysine	88.1	88.2	88.9	85.9	87.5	85.3	86.6	0.72	87.7	85.9	0.18
Methionine	84.4^b^	79.7^b^	88.7^a^	76.4^d^	79.8^c^	81.1^c^	87.1^ab^	0.36	81.8	84.1	0.30
Phenylalanine	89.5^bcd^	88.4^cd^	89.8^bcd^	86.0^d^	91.3^abc^	94.2^a^	93.3^ab^	0.46	89.0	93.7	0.01
Proline	70.2^c^	81.2^abc^	86.7^ab^	70.3^c^	77.5^bc^	85.4^ab^	92.8^a^	1.63	77.2	89.1	0.02
Serine	84.6^ab^	82.9^b^	86.2^ab^	83.6^ab^	85.2^ab^	89.9^a^	88.9^ab^	0.70	84.5	89.4	0.03
Threonine	77.7	76.0	80.2	79.2	80.2	84.1	80.4	0.95	78.6	82.3	0.35
Tyrosine	83.2^b^	82.5^b^	83.4^b^	79.8^b^	81.1^b^	88.8^a^	88.7^a^	0.57	82.0	89.7	0.001
Valine	86.0^ab^	82.3^b^	89.1^a^	76.6^c^	89.1^a^	88.5^a^	90.4^a^	0.58	84.6	89.5	0.02

^1^Quality Protein Maize.

^2^Standard error of the mean.

^3^Probability.

^abcd^different letters in the same line differ (*P* < 0.05).

#### *Standardized ileal digestibility, means comparison*

The SID of QPM 537Ig was consistently the lowest among the QPMs. The digestibility of glutamic acid and tyrosine was lower in the QPM diets than in the normal maize diets (Table 6). Methionine was less digestible in the white maize diet than in the yellow maize diet (Table 6).

## Discussion

### Energy metabolizability and nitrogen balance

The total tract digestibility of energy in the QPM diets was 1.3% lower, on average, than the normal maize diets. While urinary energy was similar between the diets, metabolizability was also lower in the QPM diets than in the white maize diet.

The starch type of QPM may explain its lower digestibility in the current study; amylose is negatively correlated with average daily gain [25]. Furthermore, the starch of waxy sorghums, which are low in amylose, is more digestible than the starch of non-waxy sorghums [26]; this has also been demonstrated with maize starch in ducks [27]. Although QPM has a vitreous endosperm phenotype, it is rich in a no-crystalline amylopectin that forms bonds with γ zein (27-kDa) [28].

The negative effect of fiber on energy digestibility [29,30] could provide another feasible explanation; dietary fiber is less digestible than other nutrients such as starch, sugars, fat, or protein (<50% vs 80-100%) [31]. Moreover, corn fiber is essentially insoluble [32]; QPM had a higher **ADF** content than the normal maize (40 g/kg QPM vs 30 g/kg maize), and this could have resulted in considerable effects on digestibility given that maize fiber, in general, is poorly digested by growing pigs [31]. Moreover, the ME:DE ratio in the current study was 0.98, which is similar to the 0.96 estimated by Noblet and van Milgen [31].

Generally, 50% of the nitrogen that is absorbed is retained in the body, and the other 50% is excreted in urine [29]. The retention of nitrogen in the current study was lower (37.5%), which could be attributed to a diet of low protein quality. Maize protein is deficient in lysine and tryptophan [3], and it is well-known that nitrogen retention in growing pigs is related to the lysine level in the diet as lysine is the first limiting AA [33]. Lysine digestibility was higher in the pigs fed QPM1 maize, and these same pigs retained 26% more nitrogen (1.7 g/d). These results are consistent with previous reports based on animal studies [1,34,35], as well as in humans where the consumption of QPM by children resulted in a 12% increase in weight [34] and a 9% increase in height and weight [36].

### Ileal digestibility

The average AA profile of the proteins in the QPM was different from that of yellow and white maize. QPM had more lysine (45%), arginine (37%), histidine (31%), glycine (23%), methionine (19%), threonine (13%), aspartic acid (13%), valine (10%), and cystine (7%) than white and yellow maize. In contrast, QPM had less leucine (-23%), alanine (-14%), phenylalanine (-13%), glutamic acid (-8%), tyrosine (-8%), and proline (-7%) than white and yellow maize. Other studies have also reported that QPM is rich in lysine [5,37,38], while the low leucine and proline content is associated with a decrease in zein protein [6].

Dietary protein content affects apparent digestibility [16,39,40]. To avoid this effect, the experimental diets were iso-nitrogenous, resulting in similar protein digestibility in all of the diets. However, the differences in amino acid content may explain the differences in digestibility. It has been previously reported that the high lysine content in QPM results in a higher AID [34,35], as was found in the current study. Similarly, the high leucine, phenylalanine, glutamic acid, alanine, tyrosine, and proline content of normal maize (white and yellow) could explain the high AID that was observed. Moreover, the low AID observed for threonine may be caused by its richness within the endogenous protein [22,41,42].

Although the use of the estimate from one endogenous protein in each experiment has been recommended to estimate the SID [39,40], it is also true that the SID has been estimated from previously published AID data and corrected with an endogenous protein that was estimated later [9,43,44] or previously [45,46]. This supports the use of a “standard” endogenous protein to correct the AID. The SID removes the effect of nutrient level on the digestibility value by correcting for basal endogenous losses [39,43,47]. The SID of lysine was similar in all of the maize diets. Additionally, when SID was estimated, threonine reached similar values to those of the other amino acids; this may be related to its richness within the endogenous protein. The SID coefficients estimated for maize in the present work were similar to those reported in previous studies [8,9,43]. However, no values for SID have been reported previously for QPM.

## Conclusions

The energy furnished by QPM was used less efficiently (metabolizability) than the energy furnished by normal maize. The AID of lysine was higher in the QPM than in the normal maize; however, the SID of lysine was similar between QPM and normal maize. The current study provides additional data about the nutrient composition, AA digestibility, and nitrogen utilization of QPM.

## Abbreviations

CIMMYT: International maize and wheat improvement centre; QPM: Quality protein maize; EF-1 α: Elongation factor 1α; DM: Dry matter; DE: Digestible energy; ME: Metabolizable energy; AA: Amino acid; ADF: Acid detergent fiber; AID: Apparent ileal digestibility; SID: Standardized ileal digestibility; ATTD: Apparent total tract digestibility.

## Competing interests

The authors declare that they have no competing interests.

## Authors’ contributions

GML conceived and designed the study; ERR carried out the lab analysis; TCRS contributed to data analysis; GML and TCRS wrote the manuscript. All authors read and approved the final manuscript.

## References

[B1] Cantarelli VS Fialho ET de Sousa RV de Freitas RTF Lima JAF Composição química, vitreosidade e digestibilidade de diferentes híbridos de milho para suínos Ciênc Agrotec 2007 31 860 864

[B2] Snow JL Stein HH Ku PK Trottier NL Amino acid digestibility and nitrogen utilization of high oil, high lysine, and waxy maize fed to growing pigs Anim Feed Sci Technol 2004 113 113 126

[B3] Jia M Wu H Clay K Jung R Larkins B Gibbon B Identification and characterization of lysine-rich proteins and starch biosynthesis genes in the opaque2 mutant by transcriptional and proteomic analysis BMC Plant Biology 2013 13 60 23586588 10.1186/1471-2229-13-60 PMC3762070

[B4] Mertz ET Bates LS Nelson OE Mutant gene that changes protein composition and increases lysine content of maize endosperm Science 1964 145 279 280 14171571 10.1126/science.145.3629.279

[B5] Nuss ET Tanumihardjo SA Quality protein maize for Africa: closing the protein inadequacy gap in vulnerable populations Adv Nutr 2011 2 217 224 22332054 10.3945/an.110.000182 PMC3090170

[B6] Wallace JC Lopes MA Paiva E Larkins BA New methods for extraction and quantitation of zeins reveal a high content of γ-zein in modified opaque-2 maize Plant Physiol 1990 92 191 196 16667246 10.1104/pp.92.1.191 PMC1062269

[B7] Habben JE Moro GL Hunter BG Hamaker BR Larkins BA Elongation factor 1α concentration is highly correlated with the lysine content of maize endosperm PNAS 1995 92 8640 8644 7567989 10.1073/pnas.92.19.8640 PMC41022

[B8] NRC Nutrient requirements of swine 1998 10 Washington, DC: The National Academy Press

[B9] INRA Tables de composition et de valeur nutritive des matières premières destinées aux animaux d’élevage. Porcs, volailles, bovins, ovins, caprins, lapins, chevaux, poissons 2002 Paris, France: Institut National de la Recherche Agronomique

[B10] International guiding principles for biomedical research involving animals The development of science-based guidelines for laboratory animal care - NCBI Bookshelfhttp://cioms.ch/publications/guidelines/1985_texts_of_guidelines.htm

[B11] Diario Oficial de la Federación Especificaciones técnicas para la producción, cuidado y uso de los animales de laboratorio. Norma Oficial Mexicana NOM-062-ZOO-1999 México, D.F. Diario Oficial de la Federación, Miércoles 2 de Agosto 2001

[B12] INRA L’alimentation des animaux monogastriques: porc, lapin, volailles 1984 Paris, France: Institut National de la Recherche Agronomique

[B13] Adeola O Lewis AJ, Southern LL Digestion and balance technique in pigs Swine Nutrition 2001 2 Boca Raton, USA: CRC Press 903 916

[B14] Le Bellego L van Milgen J Dubois S Noblet J Energy utilization of low-protein diets in growing pigs J Anim Sci 2001 79 1259 1271 11374546 10.2527/2001.7951259x

[B15] de Souza RTC Mar BB Mariscal LG Canulación de cerdos posdestete para pruebas de digestibilidad ileal: Desarrollo de una metodología Téc Pecu Méx 2000 38 143 150

[B16] Fan MZ Sauer WC Hardin RT Lien KA Determination of apparent ileal amino acid digestibility in pigs: effect of dietary amino acid level J Anim Sci 1994 72 2851 2859 7730178 10.2527/1994.72112851x

[B17] AOAC Official Methods of Analysis 2000 17 Arlington, VA. USA: Assoc. Offic. Anal. Chem

[B18] Fenton TW Fenton M An improved procedure for determination of chromic oxide in feed and feces Can J Anim Sci 1979 59 631 634

[B19] Henderson JH Ricker RD Bidlingmeyer BA Woodward C Rapid, accurate and reproducible HPLC analysis of amino acids. Amino acid analysis using Zorbax Eclipse AAA columns and the Agilent 1100 HPLC Agilent Technologies 2000 Part No5980-1193E 2000 10 Agilent technologies home page at: http://www.agilent.com/chem/supplies

[B20] Fan MZ Sauer WC Determination of apparent ileal amino acid digestibility in barley and canola meal for pigs with the direct, difference, and regression methods J Anim Sci 1995 73 2364 2374 8567474 10.2527/1995.7382364x

[B21] Furuya S Kaji Y Estimation of the true ileal digestibility of amino acids and nitrogen from their apparent values for growing pigs Anim Feed Sci Technol 1989 26 271 285

[B22] Mariscal-Landín G de Souza RTC Endogenous ileal losses of nitrogen and amino acids in pigs and piglets fed graded levels of casein Arch Anim Nutr 2006 60 454 466 17236705 10.1080/17450390600973642

[B23] Steel RGD Torrie JH Principles and procedures of statistics. A Biometrical approach 1980 2 New York: McGraw-Hill

[B24] SAS version 9.2 Statistical Analysis Systems Institute User’s guide 2008 92 SAS Institute Inc

[B25] Moore SM Stalder KJ Beitz DC Stahl CH Fithian WA Bregendahl K The correlation of chemical and physical corn kernel traits with growth performance and carcass characteristics in pigs J Anim Sci 2008 86 592 601 18073283 10.2527/jas.2007-0257

[B26] Wong JH Marx DB Wilson JD Buchanan BB Lemaux PG Pedersen JF Principal component analysis and biochemical characterization of protein and starch reveal primary targets for improving sorghum grain Plant Sci 2010 179 598 611

[B27] Zhou Z Wan HF Li Y Chen W Qi ZL Peng P Peng J The influence of the amylopectin/amylose ratio in samples of corn on the true metabolizable energy value for ducks Anim Feed Sci Technol 2010 157 99 103

[B28] Gibbon BC Wang X Larkins BA Altered starch structure is associated with endosperm modification in Quality Protein Maize PNAS 2003 100 15329 15334 14660797 10.1073/pnas.2136854100 PMC307567

[B29] Kil D Kim B Stein H Feed energy evaluation for growing pigs Asian-Aust J Anim Sci 2013 26 1205 1217 10.5713/ajas.2013.r.02 PMC4093404 25049902

[B30] Szabó C Halas V Shortcomings of the energy evaluation systems in pigs: a review Agric Conspec Sci 2013 78 153 158

[B31] Noblet J van Milgen J Energy value of pig feeds: effect of pig body weight and energy evaluation system J Anim Sci 2004 82 E-Suppl E229 E238 15471802 10.2527/2004.8213_supplE229x

[B32] Cowieson AJ Factors that affect the nutritional value of maize for broilers Anim Feed Sci Technol 2005 119 293 305

[B33] Susenbeth A Dickel T Diekenhorst A Höhler D The effect of energy intake, genotype, and body weight on protein retention in pigs when dietary lysine is the first-limiting factor J Anim Sci 1999 77 2985 2989 10568468 10.2527/1999.77112985x

[B34] Burgoon KG Hansen JA Knabe DA Bockolt AJ Nutritional value of quality protein maize for starter and finisher swine J Anim Sci 1992 70 811 817 1564005 10.2527/1992.703811x

[B35] Sullivan JS Knabe DA Bockholt AJ Gregg EJ Nutritional value of quality protein maize and food corn for starter and growth pigs J Anim Sci 1989 67 1285 1292 2737984 10.2527/jas1989.6751285x

[B36] Gunaratna NS de Groote H Nestel P Pixley KV McCabe GP A meta-analysis of community-based studies on quality protein maize Food Policy 2010 35 202 210

[B37] Azevedo RA Arruda P High-lysine maize: the key discoveries that have made it possible Amino Acids 2010 39 979 989 20373119 10.1007/s00726-010-0576-5

[B38] Zarkadas CG Yu JZ Hamilton RI Pattison PL Rose NGW Comparison between the protein quality of northern adapted cultivars of common maize and quality protein maize J Agric Food Chem 1995 43 84 93 10.1021/jf000374b 11087485

[B39] Stein HH Sève B Fuller MF Moughan PJ de Lange CFM Invited review: amino acid bioavailability and digestibility in pig feed ingredients: terminology and application J Anim Sci 2007 85 172 180 17179553 10.2527/jas.2005-742

[B40] Stein HH Fuller MF Moughan PJ Sève B Moshentin R Jansman AJM Fernandez JA de Lange CFM Deffinition of apparent, true and standardized ileal digestibility of amino acids in pigs Livest Sci 2007 109 282 285

[B41] de Souza RTC Aguilera BA Mariscal-Landín G Estimation of endogenous protein and amino acid ileal losses in weaned piglets by regression analysis using diets with graded levels of casein J Anim Sci Biotechnol 2013 4 36 24053636 10.1186/2049-1891-4-36 PMC4016253

[B42] Mariscal-Landín G Sève B Collèaux Y LeBreton Y Endogenous amino nitrogen collected from pigs with end to end ileorectal anastomosis is affected by the method of estimation and altered by dietary fiber J Nutr 1995 125 136 146 7815171 10.1093/jn/125.1.136

[B43] Yin Y-L Li T-J Huang R-L Liu Z-Q Kong XF Chu W-Y Tan B-E Deng D Kang P Yin F-G Evaluating standardized ileal digestibility in growing pigs Anim Feed Sci Technol 2008 140 385 401

[B44] Pedersen C Boisen S Establishement of tabulated values for standardized ileal digestibility of crude protein and essential amino acids in common feedstuffs for pigs Acta Agric Scand Sect A Anim Sci 2002 52 121 140

[B45] Urbaityte R Mosenthin R Eklund M Piepho HP Rademacher M Determination of standardized ileal crude protein and amino acid digestibilities in protein supplements for piglets Animal 2009 3 1696 1705 22443554 10.1017/S1751731109990474

[B46] Yin Y Gurung NK Jeaurond E Sharpe P de Lange C Digestible energy and amino acid contents in Canadian varieties of sorghum, pearl millet, high-oil corn, high-oil-high-protein corn and regular corn samples for growing pigs Can J Anim Sci 2002 82 385 391

[B47] Furuya S Kaji Y Additivity of the apparent and true digestible amino acid supply in barley, maize, wheat or soya bean based diets for growing pigs Anim Feed Sci Technol 1991 32 321 331

